# Fingolimod and Diabetic Retinopathy: A Drug Repurposing Study

**DOI:** 10.3389/fphar.2021.718902

**Published:** 2021-09-17

**Authors:** Carlo Gesualdo, Cornel Balta, Chiara Bianca Maria Platania, Maria Consiglia Trotta, Hildegard Herman, Sami Gharbia, Marcel Rosu, Francesco Petrillo, Salvatore Giunta, Alberto Della Corte, Paolo Grieco, Rosa Bellavita, Francesca Simonelli, Michele D’Amico, Anca Hermenean, Settimio Rossi, Claudio Bucolo

**Affiliations:** ^1^ Multidisciplinary Department of Medical, Surgical and Dental Sciences, University of Campania “Luigi Vanvitelli”, Naples, Italy; ^2^ “Aurel Ardelean” Institute of Life Sciences, Vasile Godis Western University of Arad, Arad, Romania; ^3^ Department of Biomedical and Biotechnological Sciences, School of Medicine, University of Catania, Catania, Italy; ^4^ Department of Experimental Medicine, University of Campania “Luigi Vanvitelli”, Naples, Italy; ^5^ Department of Ophthalmology, University of Catania, Catania, Italy; ^6^ Pharmacy Department, University of Naples Federico II, Naples, Italy; ^7^ Department of Histology, Faculty of Medicine, Vasile Goldis Western University of Arad, Arad, Romania

**Keywords:** fingolimod, sphingosine 1-phosphate receptor, melanocortin receptor 1, melanocortin receptor 5, diabetic retinopathy

## Abstract

This study aimed to investigate the interactions between fingolimod, a sphingosine 1-phosphate receptor (S1PR) agonist, and melanocortin receptors 1 and 5 (MCR1, MCR5). In particular, we investigated the effects of fingolimod, a drug approved to treat relapsing-remitting multiple sclerosis, on retinal angiogenesis in a mouse model of diabetic retinopathy (DR). We showed, by a molecular modeling approach, that fingolimod can bind with good-predicted affinity to MC1R and MC5R. Thereafter, we investigated the fingolimod actions on retinal MC1Rs/MC5Rs in C57BL/6J mice. Diabetes was induced in C57BL/6J mice through streptozotocin injection. Diabetic and control C57BL/6J mice received fingolimod, by oral route, for 12 weeks and a monthly intravitreally injection of MC1R antagonist (AGRP), MC5R antagonist (PG20N), and the selective S1PR1 antagonist (Ex 26). Diabetic animals treated with fingolimod showed a decrease of retinal vascular endothelial growth factor A (VEGFA) and vascular endothelial growth factor receptors 1 and 2 (VEGFR1 and VEGFR2), compared to diabetic control group. Fingolimod co-treatment with MC1R and MC5R selective antagonists significantly (*p* < 0.05) increased retinal VEGFR1, VEGFR2, and VEGFA levels compared to mice treated with fingolimod alone. Diabetic animals treated with fingolimod plus Ex 26 (S1PR1 selective blocker) had VEGFR1, VEGFR2, and VEGFA levels between diabetic mice group and the group of diabetic mice treated with fingolimod alone. This vascular protective effect of fingolimod, through activation of MC1R and MC5R, was evidenced also by fluorescein angiography in mice. Finally, molecular dynamic simulations showed a strong similarity between fingolimod and the MC1R agonist BMS-470539. In conclusion, the anti-angiogenic activity exerted by fingolimod in DR seems to be mediated not only through S1P1R, but also by melanocortin receptors.

## Introduction

Fingolimod, an analog of myriocin (Mehling et al., 2011), is a sphingosine 1-phosphate receptors agonist (S1PRs), and it is used in monotherapy for the treatment of relapsing-remitting multiple sclerosis (RR-MS) (Cohen et al., 2010; Kappos et al., 2010). The modulation of the S1P1R activity could be useful for treatment of several diseases that share immune-inflammatory pathogenic mechanisms, such as rheumatoid arthritis, fibrosis, choroidal neovascularization (CNV), and diabetic retinopathy (DR) (Bing et al., 2009; Mehling et al., 2011; Yoshida et al., 2013; Fan and Yan, 2016). To this regard, recent studies have shown a protective role of fingolimod in a rat model of DR induced by intraperitoneal injection of streptozotocin (STZ): this anti-inflammatory action was exerted by a reduction of pro-inflammatory cytokines and molecules of adhesion to the vessel wall (Fan and Yan, 2016). Moreover, fingolimod was able to reduce vascular permeability, increasing tight junctions expression in the blood retinal barrier (Fan and Yan, 2016). Additionally, several studies reported a preserved macular structure and thickness over time in RR-MS patients treated with fingolimod (Fruschelli et al., 2019; d’Ambrosio et al., 2020). Worthy of note, although macular edema is reported as a side effect of fingolimod administration with an incidence of 0.3–1.2% (Nolan et al., 2013), two studies evidenced that RR-MS patients treated with fingolimod did not show any case of macular edema (Fruschelli et al., 2019; Rossi et al., 2020).

We have previously shown that activation of melanocortin receptors 1 and 5 (MC1R and MC5R) reduced retinal damage in mouse model of DR, preventing alterations of blood retinal barrier and reducing local pro-inflammatory and pro-angiogenic mediators such as cytokines, chemokines, and vascular endothelial growth factor (VEGF) (Maisto et al., 2017; Rossi et al., 2021). Interestingly, recent evidence showed that S1PR1 with melanocortin signaling pathway can play an important role in the regulation of energy homeostasis of hypothalamic neurons in rodents (Silva et al., 2014). With a virtual screening approach aimed at drug repurposing in DR, we first identified fingolimod as putative ligand for MC1R and MC5R. Indeed, we hypothesized that the interplay between sphingosine pathway and melanocortin pathway could also occur at the level of ocular structures. Therefore, we hypothesized that fingolimod may be protective in retinal degenerative diseases, such as DR, through binding at melanocortin receptors. Therefore, in the present study we investigated the interaction between fingolimod and melanocortin receptors in an animal model of DR, using pharmacological tools such as selective MC1R and MC5R antagonists.

## Materials and Methods

### Molecular Modeling

Structural models of human melanocortin receptor 1 (hMC1R) and human melanocortin receptor 5 (hMC5R) were built with the Advanced Homology Modeling task of Schrödinger Maestro, using as primary sequences for hMC1R and the hMC5R, FASTA files from accession numbers Q01726.2 and NP_005904.1, respectively. Both models were built using as a template the x-ray structure of human melanocortin receptor 4 (PDB:6W25); because the Advance Homology Modeling Task gave, for this template, the highest scores for both the hMC1R (score 437, identity 43%, homology 60%, gaps 5%) and hMC5R (score 591, identity 60%, homology 75%, gaps 4%). The structural optimization (Prime energy minimization) of hMC1R and hMC5R led to similar structural models at least in the transmembrane domain (RMSD = 0.463Å). Therefore, as previously shown (Platania et al., 2012), in order to differentiate the two structural models, we carried out molecular dynamics simulation of hMC1R and hMC5R in an explicit water-membrane system, with Desmond Molecular Dynamics Simulation Task of Schrödinger Maestro. Specifically, an orthorhombic box has been created, the receptors were included in a 30 Å^3^ POPC lipid membrane-water system according to output from OMP database (https://opm.phar.umich.edu/). TIP3P water molecules were added to the system, along with NaCl (150 mM). After membrane protein equilibration protocol, 6 ns NPγT ensemble production runs were carried out. After simulations of the two membrane receptor systems, molecular dynamics has been clustered in five clusters by means of Desmond Trajectory Analysis Clustering Task, based on RMSD values and applying the cut-off of 10 for frequency value. Therefore, we built for each 10 clusters (5 for hMC1R and 5 for hMC5R) a grid centered on pocket identified by SiteMap task. The grid was built tacking into account the peptide docking option. After that, selective active MCxR ligands were docked with Glide docking task, by taking advantage of ensemble docking option (i.e., multiple rigid receptor conformations). Ligand-receptor complexes were rescored with application of MM-GBSA calculation. Specifically, for MM-GBSA calculation, residues within 15 Å from ligands were free to move during minimization protocol, applying an implicit solvation and membrane model, according to protocol already published (Stark et al., 2020). Agouti related protein (AGRP) structure, an MC1R antagonist, was retrieved from the PDB: 1MR0, and subjected to energy minimization in implicit water model with Prime (Schrodinger Maestro). The BMS-470539 (BMS) is a MC1R agonist, and its 2D structure was built with the webserver https://cactus.nci.nih.gov/translate/. The. sdf files for two macrocycles PG901 (MC5R agonist) and PG20N (MC5R antagonist) were also built with https://cactus.nci.nih.gov/translate/. All ligands were then subjected to the LigPrep task and the ionization state was assigned at pH 7.4. Macrocycle conformation sampling task was applied to AGRP, PG901, and PG20N ligands, with the following settings: GB-SA electrostatic model, OPLS3e force-field, 5,000 simulation cycles, 5,000 Macrocycle specific LLMOD search step, and enhanced torsion sampling.

This preliminary docking step was used to rescore receptor clusters, on the basis of the docking scores and predicted ΔG_binding_ energy, relative to selective hMC1R and hMC5R ligands. After that, we carried out virtual screening of drugs already approved for several indications (Food and Drug Administration–FDA-approved drugs database), according to our previous published protocol (Platania et al., 2020).

Fingolimod/hMC1, fingolimod/hMC5, BMS/hMC1, PG901/hMC5, AGRP/hMC1, and PG20N/hMC5 complexes were built through the molecular docking step as described above. Therefore, molecular dynamics simulations in explicit POPC membrane and TIP3P water were carried out as follows: membrane equilibration steps and 20 ns production runs, applying the same protocol described above for the unbound hMC1 and hMC5 receptors. Simulation Interaction task, within Schrödinger maestro environment, was used, providing information regarding ligand-receptor interactions. Salt-bridges of hMC1R and hMC5R ligand complexes were also analyzed with Visual Molecular Dynamics software (VMD version 1.9.3) (Humphrey et al., 1996). Differences between contact maps of ligand-receptor complexes, generated with Schrödinger Maestro, were analyzed applying Fuzzy Logic algorithm through access to the web server (https://online-image-comparison.com/). The fuzz option was set to 4 as cut-off value, to highlight the differences between contact maps.

### Compounds

Fingolimod (FTY720) was purchased from MedChemExpress (Italy, catalog number HY-12005/CS-0114); Ex 26 [1-(5'-((1-(4-chloro-3-methylphenyl)ethyl)amino)-2′-fluoro-3,5-dimethyl-[1,1′-biphenyl]-4-ylcarboxamido cyclopropanecarboxylic acid)], a selective S1PR1 antagonist, from Tocris (Italy, catalog number 5833) and STZ from Santa Cruz Biotechnology (Italy, catalog number sc-200719). AGRP and PG20N, used respectively as MCR1 and MCR5 antagonists, were synthetized as previously described (Carotenuto et al., 2015; Merlino et al., 2018; Merlino et al., 2019).

### Animals and Experimental Design

Animal care and experimental procedures were approved by the Institutional Ethical Committee of the “Vasile Goldis” Western University of Arad (number, 29/May 17, 2017) and were in line with the Association for Research in Vision and Ophthalmology (ARVO) Statement for the Use of Animals in Ophthalmic and Vision Research. Six-week-old C57BL/6J male mice (22.5 ± 1.6 g) (Cantacuzino National Research Institute of Bucharest, Romania) were housed in single standard cages with ad libitum access to mineral water and standard chow. They were exposed to 12 h light/12 h dark cycle, controlled humidity, and temperature. After an overnight fast, mice were intraperitoneally (i.p.) injected with a single dose of STZ (65 mgkg−1 of body weight) freshly dissolved in 50 mM sodium citrate buffer (pH 4.5) (STZ group) or with sodium citrate buffer alone as controls (CTR group). After 4 h fasting, a one-touch glucometer (Accu Chek Active, Roche Diagnostics, United States) was used to measure blood glucose levels. STZ mice showing fasting blood glucose levels higher than 2.5 gl−1 on two consecutive weeks were included in the experimental design as type 2 diabetic mice. Mice were randomized into the following experimental groups (N = 5 per group): I. control non-diabetic mice (CTR group); II. diabetic mice (STZ group) receiving intravitreally sterile phosphate saline buffer (PBS, p-H. 7.4); III. diabetic mice receiving per *os* fingolimod (STZ + Fingolimod group); IV. diabetic mice receiving per *os* fingolimod and intravitreally MC1 receptor antagonist AGRP (STZ + Fingolimod + AGRP group); V. diabetic mice receiving per *os* fingolimod and intravitreally PG20N (MC5R antagonist) (STZ + Fingolimod + PG20N group); VI. diabetic mice receiving per *os* fingolimod and intravitreally Ex 26 (selective S1PR1 receptor antagonist) (STZ + Fingolimod + Ex 26 group). Particularly, after 2 weeks from STZ injection, fingolimod was orally administered for 12 weeks at a dose of 0.3 mg/kg/day, contained in 20 ml of drinking water as calculated daily for each mouse intake (Bonfiglio et al., 2017). PBS, AGRP (14.3 µM in sterile PBS) (Rossi et al., 2021), PG20N (130 nM in sterile PBS) (Rossi et al., 2021), and Ex 26 (3 mg/kg in sterile PBS) (Cahalan et al., 2013) were administered by intravitreal injections (5 µL). These were performed after 2 weeks from STZ injection in mice with blood glucose levels higher than 2.5 gl−1 (baseline), then after 4 and 8 weeks.

### Intravitreal Injections

To perform intravitreal injections, mice were anesthetized by pentobarbital (45 mg/kg in saline). To induce dilatation of pupils, tropicamide (5%) was instilled into the right eye of each animal plus tetracaine (1%) for local anesthesia. PBS, AGRP, PG20N, and EX 26 preparations (5 μL volume) were administered intravitreally into the right eye using a sterile syringe fitted with a 30-gauge needle (Microfine; Becton Dickinson AG, Meylan, France) (Rossi et al., 2021). Before the intravitreal injection, an anterior chamber paracentesis of similar volume was performed to avoid an increase of the intraocular pressure (Biswas et al., 2007).

### Fluorescein Angiography

FA was performed by using a Topcon TRC-50DX apparatus (Topcon, Tokyo, Japan) after intraperitoneal injection of 10% fluorescein sterile solution (1 ml/kg body weight, AK-Fluor; Akorn, Inc.). To display the retinal vasculature and to evaluate the early DR typical alterations, C57BL/6J animals were monitored by FA over a 12-week period, with specific analyses at baseline and at weeks 4, 8, and 12. Particularly, mice were consecutively labelled from 1 to 5 in each group, to repeat FA to the same animal at each time point of the study. Vessel abnormalities (VA) were graded from 0 to 4 according to the following score: 0 = absence of vessel abnormalities; 1 = vessel thinning; 2 = vessel thinning and tortuosity; 3 = vessel thinning, tortuosity, and/or crushing; 4 = vessel thinning and tortuosity, venous beading, rosary-like vessels. The score was reported as a mean of the vascular alterations observed at the different time points during the follow-up. VA were scored by two different ophthalmologists (always the same) unaware of group labeling. At the end of the follow-up, animals were sacrificed and retina were dissected, placed in cooled PBS, then fixed by immersion in 10% neutral buffered formalin and paraffin-embedded for immunohistochemistry (Rossi et al., 2021).

### Immunohistochemistry

The primary antibodies used for the immunohistochemical studies were the rabbit polyclonal anti-Vascular Endothelial Growth Factor Receptor 1 (VEGFR1) (ab32152, Abcam, United Kingdom) and anti-Vascular Endothelial Growth Factor Receptor 2 (VEGFR2) (ab2349, Abcam, United Kingdom). Eye sections of 5 μm thickness were deparaffinized in Bond Dewax solution (Leica Biosystems, Germany) and rehydrated prior to epitope retrieval in Novocastra Epitope Retrieval solution (Leica Biosystems, Germany) (just in case of VEGFR1). After 10 min incubation with 3% H2O2, followed by the blocking solution (Novocastra Leica Biosystems, Germany) also for 10 min, the tissue sections were incubated overnight at 4 °C with anti-VEGFR1 and anti-VEGFR2 antibodies (1:100 dilution). Detection was performed using a polymer detection system (RE7280-K, Novolink max Polymer detection system, Novocastra Leica Biosystems) and 3,3′- diaminobenzidine (DAB, Novocastra Leica Biosystems) as chromogenic substrate, according to the manufacturer’s instructions. Hematoxylin staining was applied before dehydration and mounting. Negative controls included substitution of the first antibody with normal rabbit serum. Images were acquired by light microscopy (Olympus BX43, Hamburg, Germany).

### Enzyme-Linked Immunosorbent Assay

Vascular Endothelial Growth Factor A (VEGFA) levels were assessed in retinal lysates obtained from an adjunctive experimental set, in order to confirm IHC data on VEGFR1 and VEGFR2 expression. C57BL/6J male mice (N = 5 per group) were treated as previously described in section 2.3 and 2.4, by receiving bilateral intravitreal injections (N = 10 retinas per group). VEGFA levels were detected in retinal lysates by ELISA (MBS704351, MyBiosource, San Diego, CA, United States), following the manufacturer’s instructions for tissue homogenates.

### Statistical Analysis

Investigators that carried out FA and immunohistochemistry and ELISA analyses were blind to group labels. After graph design and rough statistical analysis, labels were unveiled by principal investigators. Statistical significance was assessed by one-way ANOVA, followed by Tukey’s multiple comparisons test by using GraphPad Prism v.6 (GraphPad Software, La Jolla, CA, United States). Differences were considered statistically significant for *p* values <0.05.

## Results

### Virtual Screening in Search of MC1R and MC5R Agonists, Repurposing of FDA Approved Drugs

Structural models of human MC1R and MC5R receptors were built with the Advanced Molecular modeling task of Schrödinger Maestro. Before virtual screening protocol, we carried out 6 ns molecular dynamics of hMC1 and hMC5 receptors embedded in an explicit water-membrane models. Then, we clustered MD trajectories on the basis of c-alpha carbons root mean square deviation (RMSD), then we carried out molecular docking of validated hMC1R and hMC5R agonists and antagonists (Merlino et al., 2019), in order to re-score the structural clusters of hMC1R and hMC5R, to be used for virtual screening of FDA approved drugs. Through the virtual screening approach, we identified several compounds (encoded with partial ATC codes) with putative activity on melanocortin receptors: diuretics (C03), anti-diabetic drugs (A10), anti-neoplastic agents (L01), anti-protozoal (P01), anti-hemorrhagic (B02), antibiotics and chemotherapeutics (D06), and anti-bacterial agents (J01). Interestingly, fingolimod (L04AA27) had the best scores for both hMC1R and hMC5R (Table 1); therefore, we tested the effects of fingolimod (FTY720) intravitreal administration in an *in vivo* model of diabetic retinopathy.

**TABLE 1:** Virtual screening of FDA approved compounds to be repurposed as melanocortin ligands. Bold text within the table is referred to the predicted binding free energy of fingolimod, respectively to hMC1R and to hMC5R, as reported in round brackets.

Selective ligands	Re-score on hMCxR model kcal/mol (receptor)
BMS-470539	−85.2 (hMC1R)
AGRP	−197.9 (hMC1R)
PG901	−111.0 (hMC5R)
PG20N	−115.7 (hMC5R)
**ATC of compounds**	**ΔG _binding_ Kcal/mol (receptor)**
C03	−41.5 (hMC1R)
A10	−45 (hMC1R)
L01	−49.6 (hMC1R)
L01	−23 (hMC1R)
P01	−17 (hMC1R)
B02	−13 (hMC1R)
D06	−34 (hMC5R)
**L04AA27**	**−77 (hMC1R)**
	−**85 (hMC5R)**
J01	−50 (hMC5R)
A10	−66 (hMC5R)

### Retinal Vascular Abnormalities Evidenced by FA Analysis

**FIGURE 1:**
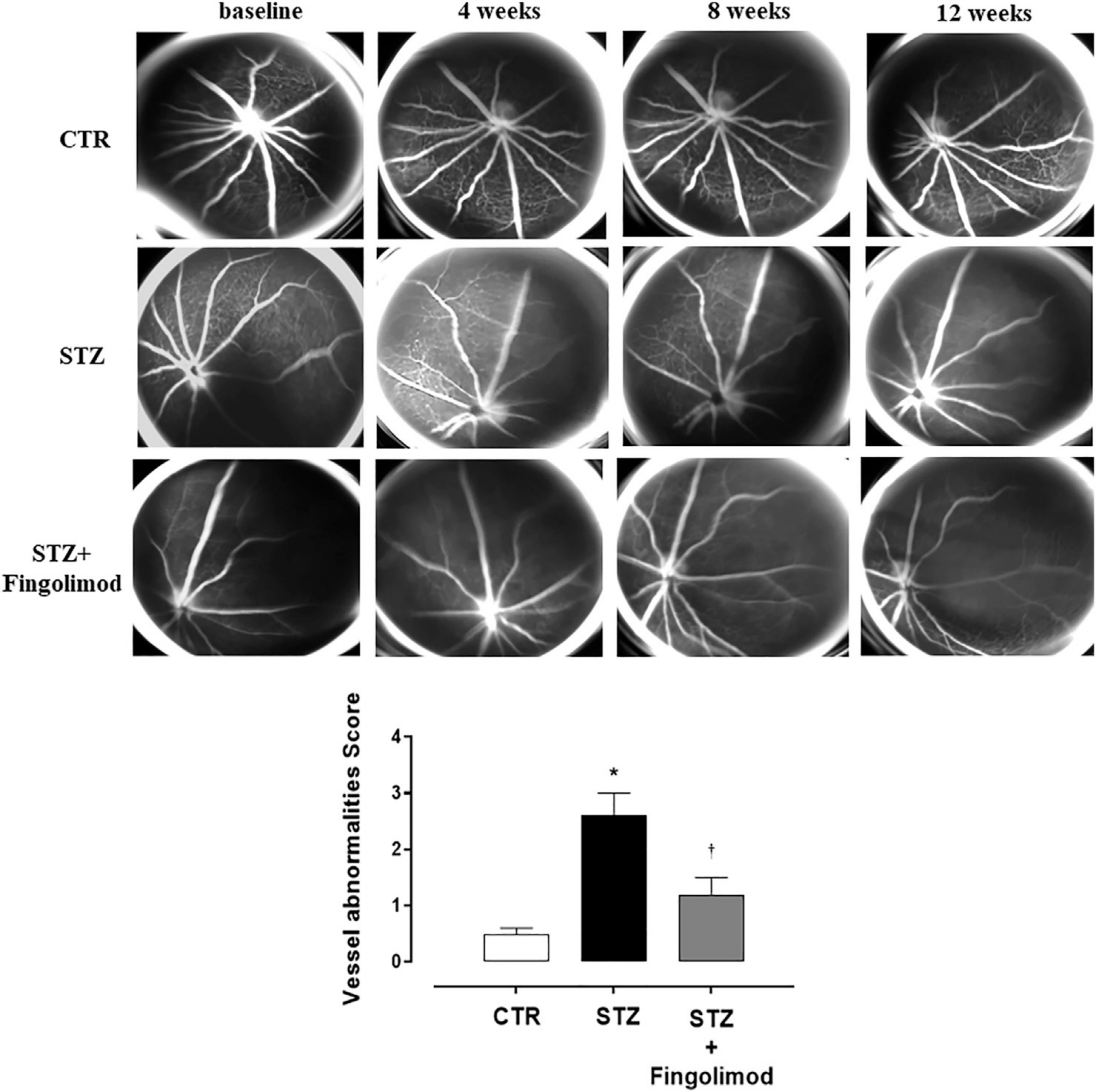
Representative FA images of eyes of non-diabetic mice (CTR), diabetic mice (STZ), and diabetic mice treated with Fingolimod (STZ + Fingolimod) during the follow-up. In CTR mice no changes in retinal vascularity were noticed during follow-up. Instead, in STZ mice, there was an increase in the irregularity of the vessel size, which began at 4 weeks and looked appeared more evident at 8 weeks, in which a pattern of the “rosary-like” vessel was appreciated (red arrow). At 12 weeks there was a further thinning of the vascular caliber. In the STZ + Fingolimod group, no significant changes in retinal vascularity were seen during follow-up. Vessel abnormalities score (graded from 0 to 4) was calculated as the average of the vascular alterations observed (N = 5 animals per group). Vessel abnormalities were graded from 0 to 4 based on the presence of vessel thinning, tortuosity, venous beading, and rosary-like vessels. Each image represents the same retina of the same mouse but at different time points (at baseline, 4–12 weeks of treatment). Statistical significance was assessed by one-way ANOVA, followed by Tukey’s multiple comparison test. *p < 0.05 vs CTR; ^†^ *p* < 0.05 *vs* STZ.

Three out of five eyes per group showed severe (2–4 score) retinal vascular abnormalities (VA) at FA exam. Particularly, an initial irregularity of the vessel size in diabetic mice (STZ group) was evident starting from 4 weeks. This became progressively more accentuated and associated with a vessel thinning both at 8 and 12 weeks. VA mean observed in STZ group was 2.6 ± 0.4 (*p* < 0.01 vs CTR) (Figure 1).

Similar to control group (CTR non-diabetic mice), no significant changes in retinal vascularity were observed during follow-up in diabetic mice treated with fingolimod (STZ + Fingolimod), which showed a VA score of 1.2 ± 0.3 (*p* < 0.05 vs STZ) (Figure 1).

**FIGURE 2:**
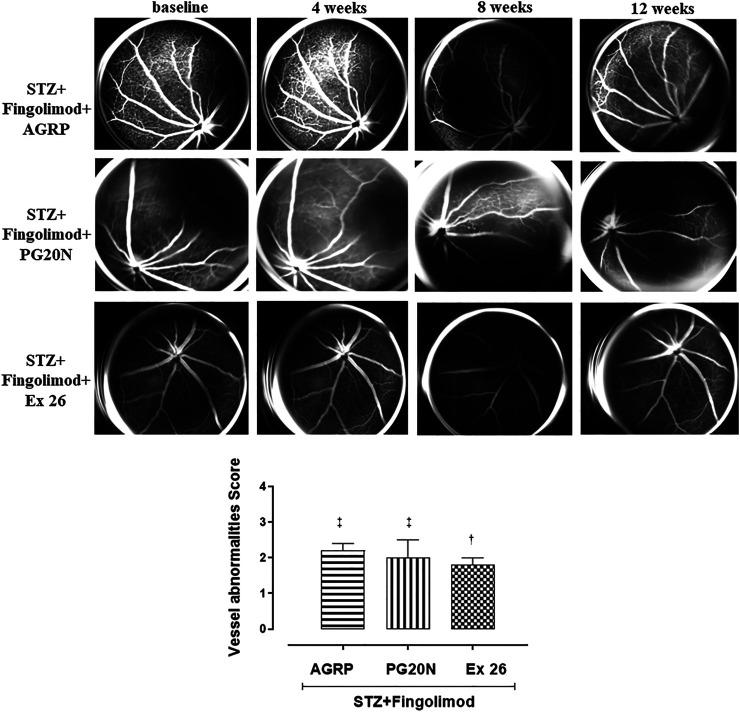
Representative FA images of eyes from diabetic mice treated with Fingolimod and MC1R antagonist (STZ + Fingolimod + AGRP), MC5R antagonist (STZ + Fingolimod + PG20N), and SP1R1 antagonist (STZ + Fingolimod + Ex 26) during the follow-up. The STZ + Fingolimod + AGRP mice showed irregularity of the vessel size, which did not significantly change over time. The STZ + Fingolimod + PG20N group showed a slight progressive thinning of the vascular caliber during the follow-up. In the STZ + Fingolimod + Ex 26 group, neither the appearance of typical signs of RD nor a significant variation of the size or of the vascular course was appreciated, during the follow up. Vessel abnormalities score (graded from 0 to 4) was calculated as the average of the vascular alterations observed (N = 5 animals per group). Vessel abnormalities were graded from 0 to 4 based on the presence of vessel thinning, tortuosity, venous beading, and rosary-like vessels. Each image represents the same retina of the same mouse but at different time points (at baseline, 4–12 weeks of treatment). Statistical significance was assessed by one-way ANOVA, followed by Tukey’s multiple comparison test. ^†^ *p* < 0.05 *vs* STZ; ^‡^ *p* < 0.05 *vs* STZ + Fingolimod.

Diabetic mice (STZ + Fingolimod + AGRP group) treated with fingolimod and AGRP, a MC1R antagonist, showed irregularity in vessel morphology and modification of vessel size, which did not significantly change over time. Diabetic mice (STZ + Fingolimod + PG20N group), treated with both fingolimod and PG20N, a MC5R antagonist, showed a slight progressive thinning of the vascular caliber at various time points. The above-mentioned experimental groups showed a VA score significantly higher compared to STZ + Fingolimod mice (STZ + Fingolimod + AGRP = 2.2 ± 0.2; STZ + Fingolimod + PG20N = 2.0 ± 0.5, both *p* < 0.05 *vs* STZ + Fingolimod) (Figure 2).

On the contrary, in diabetic mice receiving fingolimod in combination with the selective S1P1R antagonist (STZ + Fingolimod + Ex 26 group), neither the appearance of DR typical signs nor a significant variation of the size, or of the vascular course was appreciated during the follow up. This was confirmed by the VA score, which was reduced to 1.8 ± 0.2 (*p* < 0.05 *vs* STZ) (Figure 2).

### VEGFR1 and VEGFR2 Expression

**FIGURE 3:**
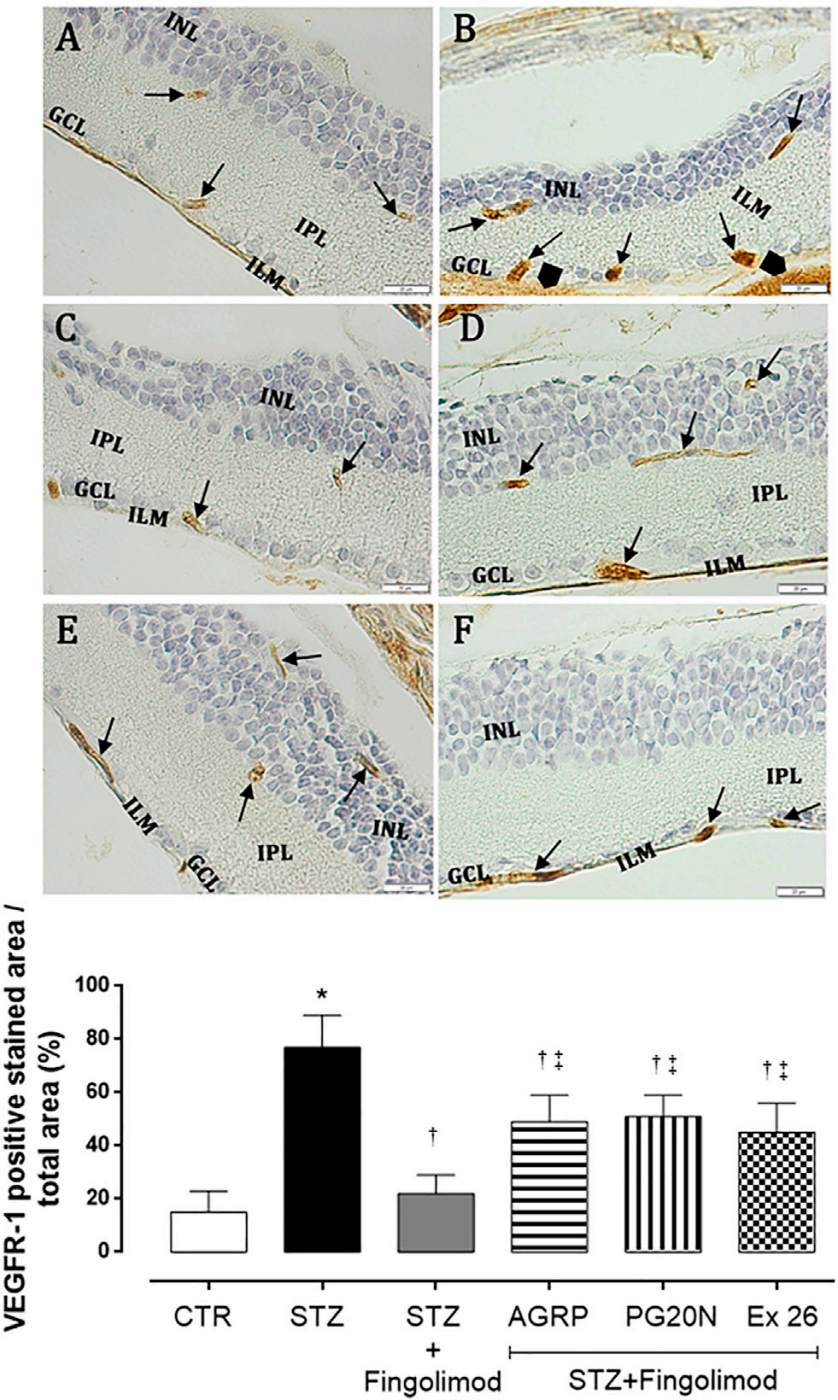
VEGFR1 immunohistochemistry in the retina of non-diabetic mice [panel **(A)**, CTR], diabetic mice [panel **(B)** STZ], diabetic mice receiving Fingolimod alone [panel **(C)**, STZ + Fingolimod], or in combination with MC1R antagonist [panel **(D)**, STZ + Fingolimod + AGRP], MC5R antagonist [panel **(E)**, STZ + Fingolimod + PG20N], and SP1R1 antagonist [panel **(F)**, STZ + Fingolimod + Ex 26]. VEGFR1 positive stain (arrow). VEGFR1 protein levels are reported as percentage (%) ± standard deviation (S.D.) of positive stained area/total area. The images are representative of 10 histological observations per group. Statistical significance was assessed by one-way ANOVA, followed by Tukey’s multiple comparison test. *p < 0.05 vs CTR; ^†^ *p* < 0.05 vs STZ; ^‡^ *p* < 0.05 vs STZ + Fingolimod. INL, inner nuclear layer; IPL, inner plexiform layer; ILM, inner limiting membrane; GLC, ganglion cell layer; retinal microvessels staining of VEGFR1 (arrow), non-vascular staining of VEGFR-1 (arrowhead); magnification: 40X; scale bar: 20 µm. N = 5 retinas per group.

VEGFR1 was expressed in all microvascular structures that were positive in retinas of both control non-diabetic (CTR group; 15 ± 8% expressing VEGFR1) and diabetic retinas (STZ group; 77 ± 12% expressing VEGFR1; *p* < 0.05 *vs* CTR) (Figures 3A,B, respectively). Staining was more intense in retinal microvessels of STZ mice, which displayed a hypertrophic morphology, compared to control retinas. In all diabetic retinas, granular VEGFR1 staining was also observed outside the retinal vasculature, in the inner limiting membrane (IML) (Figure 3B). Instead, the expression of VEGFR1 in the diabetic mice receiving fingolimod (STZ + Fingolimod group) was close to the control (22 ± 7% expressing VEGFR1; *p* < 0.05 vs STZ) (Figure 3C). Co-administration of fingolimod with either with MC1R or MC5R antagonists (STZ + Fingolimod + AGRP and STZ + Fingolimod + PG20N groups) led to a higher VEGFR1 immunostaining in the ganglion cell layer (GCL) and the inner plexiform and nuclear layer (INL), compared to STZ + Fingolimod treated group, but VEGFR1 staining was reduced compared to STZ group (49 ± 10% and 51 ± 8% expressing VEGFR1, respectively; both *p* < 0.05 vs STZ and *p* < 0.05 vs STZ + Fingolimod) (Figures 3D,E). In the retina of diabetic mice administered with fingolimod and SP1R1 selective antagonist (STZ + Fingolimod + Ex 26), some VEGFR1 positive retinal ganglion cells and amacrine and bipolar cells were detected (45 ± 11% expressing VEGFR1; *p* < 0.05 vs STZ and *p* < 0.05 vs STZ + Fingolimod) (Figure 3F).

**FIGURE 4:**
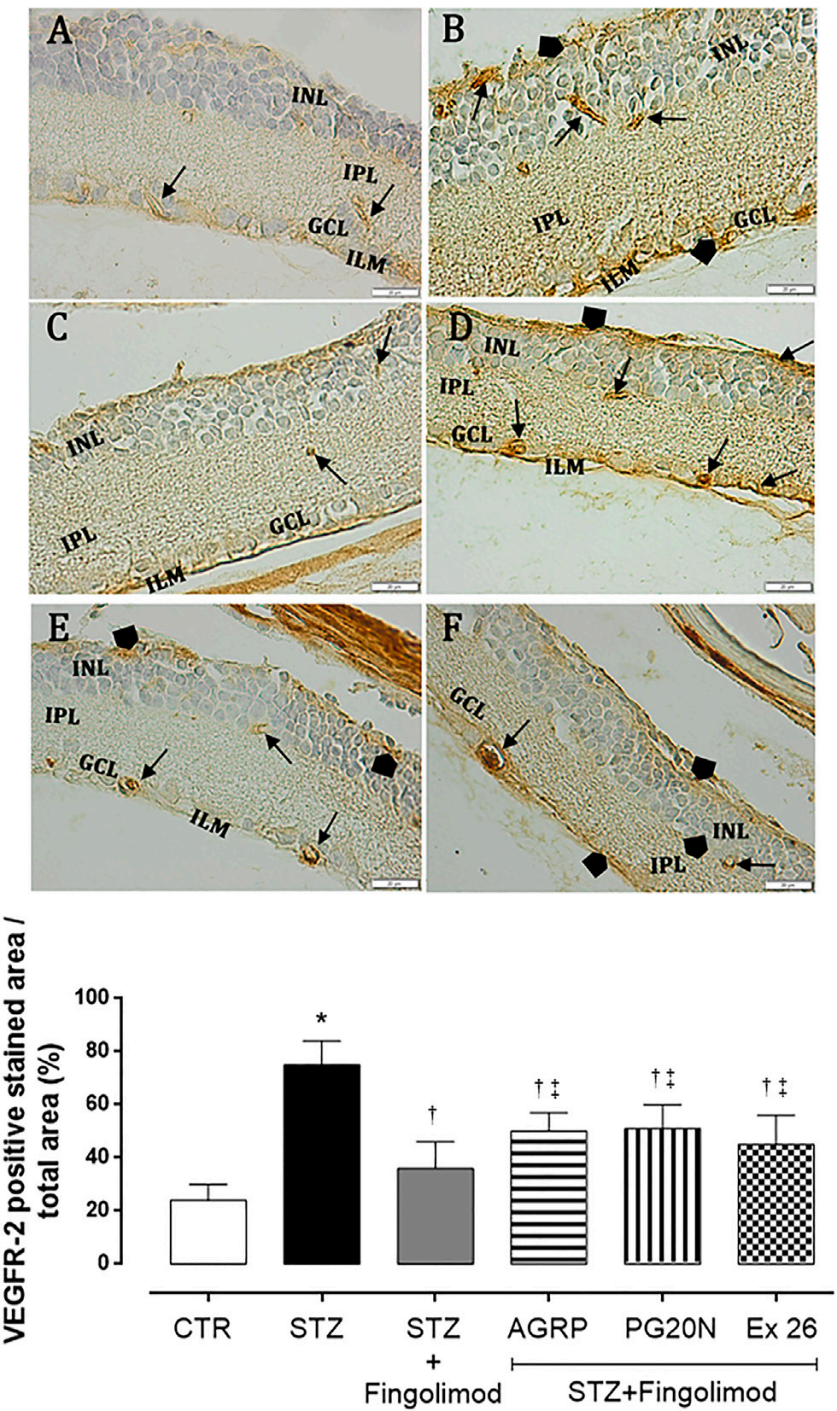
VEGFR2 immunohistochemistry in the retina of non-diabetic mice [panel **(A)**, CTR], diabetic mice [panel **(B)**, STZ], diabetic mice receiving fingolimod alone [panel **(C)**, STZ + Fingolimod] or in combination with MC1R antagonist [panel **(D)**, STZ + Fingolimod + AGRP], MC5R antagonist [panel **(E)**, STZ + Fingolimod + PG20N], and SP1R1 antagonist [panel **(F)**, STZ + FTY720+Ex 26]. VEGFR2 positive stain (arrow). VEGFR2 protein levels are reported as percentage (%) ± S.D. of positive stained area/total area. The images are representative of 10 histological observations per group. Statistical significance was assessed by one-way ANOVA, followed by Tukey’s multiple comparison test. *p < 0.05 vs CTR; ^†^ *p* < 0.05 vs STZ; ^‡^ *p* < 0.05 vs STZ + Fingolimod. INL, inner nuclear layer; IPL, inner plexiform layer; ILM, inner limiting membrane; GLC, ganglion cell layer; retinal microvessels staining of VEGFR-2 (arrow), non-vascular staining of VEGFR-2 (arrowhead); magnification 40X; scale bar: 20 µm. N = 5 retinas per group.

Staining of VEGFR2 was weak in retina of control mice, with a patchy distribution pattern in retinal microvessels (CTR group; 24 ± 6% expressing VEGFR2) (Figure 4A). In diabetic retina (STZ group), staining of VEGFR-2 was observed in microvessels within the ganglion cell layer (GCL), inner plexiform (IPL), and nuclear layer (INL). Additionally, VEGFR2 granular staining was detected also in the non-vascular areas such as inner limiting membrane (ILM) and the outer part of the inner nuclear layer (INL) (75 ± 9% expressing VEGFR2; *p* < 0.05 vs CTR) (Figure 4B). Diabetic mice administered with fingolimod (STZ + Fingolimod) showed weak VEGFR2 retinal immunopositivity, similar to control (36 ± 10% expressing VEGFR2; *p* < 0.05 vs STZ) (Figure 4C). In the inner limiting membrane (ILM), ganglion cell layer (GCL) and the outer part of the inner nuclear layer (INL), retinas of groups STZ + Fingolimod + AGRP and STZ + Fingolimod + PG20N showed a VEGFR2 staining higher compared to diabetic mice treated only with fingolimod, but lower compared to STZ untreated mice (*p* < 0.05 vs STZ + Fingolimod and *p* < 0.05 vs STZ) (Figures 4D,E). Similarly, VEGFR2 labeling and localization was evidenced in diabetic mice treated with fingolimod and SP1R1 selective antagonist (STZ + Fingolimod + Ex 26; 45 ± 11% expressing VEGFR2, *p* < 0.05 vs STZ + Fingolimod and *p* < 0.05 vs STZ) (Figure 4F).

### VEGFA Levels

**FIGURE 5:**
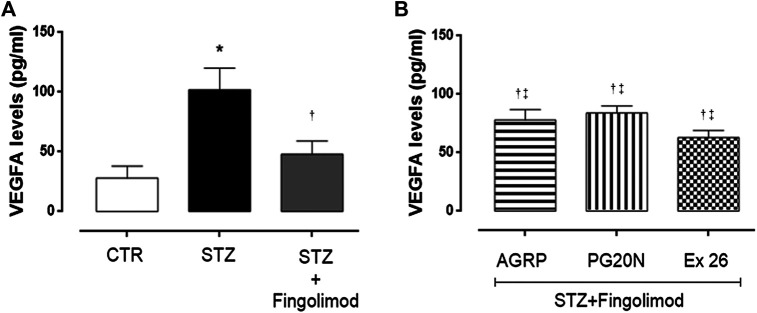
VEGFA levels in retina of non-diabetic mice [panel **(A)**, CTR], diabetic mice [panel **(A)**, STZ], diabetic mice receiving fingolimod alone [panel **(A)**, STZ + Figolimod], or in combination with MC1R antagonist [panel **(B)**, STZ + Fingolimod + AGRP], MC5R antagonist [panel **(B)**, STZ + Fingolimod + PG20N], and SP1R1 antagonist [panel **(B)**, STZ + FTY720+Ex 26]. VEGFA levels, assayed by ELISA, are reported as pg/ml ± S.D. Statistical significance was assessed by one-way ANOVA, followed by Tukey’s multiple comparison test. *p < 0.05 vs CTR; ^†^ *p* < 0.05 vs STZ; ^‡^ *p* < 0.05 vs STZ + Fingolimod. N = 10 retinas per group.

VEGFR1 and VEGFR2 retinal immunostaining results were confirmed by retinal VEGFA levels assessment through ELISA. Particularly, the highest VEGFA levels were detected in diabetic retina (102 ± 8 pg/ml; *p* < 0.01 vs CTR group), while levels were significantly reduced by fingolimod treatment (48 ± 4 pg/ml; *p* < 0.01 vs STZ) (Figure 5A). The combination of fingolimod plus MC1R or MC5R antagonists significantly increased VEGFA levels compared to fingolimod alone (78 ± 9 pg/ml and 84 ± 6 pg/ml, both *p* < 0.05 vs STZ + Fingolimod), while diabetic mice receiving fingolimod and SP1R1 selective blocker exhibited intermediate VEGFA levels between STZ and STZ + Fingolimod groups (63 ± 6 pg/ml, *p* < 0.05 *vs* STZ) (Figure 5B).

### Molecular Dynamics

**FIGURE 6:**
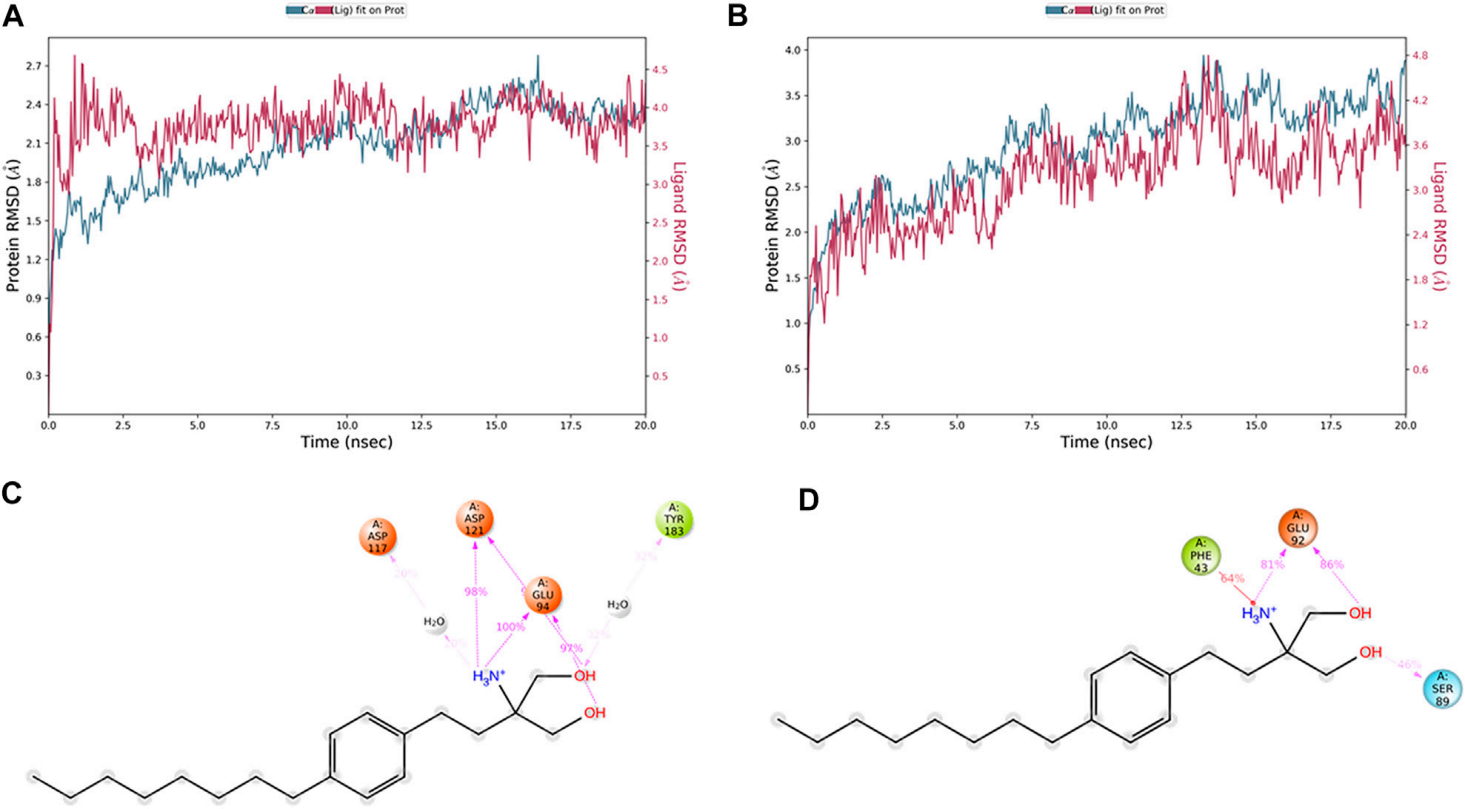
Fingolimod stably bound to hMC1R and hMC5R during 20 ns MD simulation. **(A)** root mean square deviation (RMSD) of hMC1R (blue plot) and fingolimod (red blot) during 20 ns of MD simulation, of the complex embedded in a POPC membrane. **(B)** RMSD of hMC5R (blue plot) and fingolimod (red blot) during 20 ns of MD simulation, of the complex embedded in a POPC membrane. **(C)** Fingolimod interactions with hMC1R, and frequency (%) during 20 ns MD simulation. **(D)** Fingolimod interactions with hMC5R, and frequency (%) during 20 ns MD simulation.

The *in vivo* pharmacological studies, through co-administration of selective melanocortin antagonists, evidenced that fingolimod exerted anti-angiogenic effects also by activation of MC1R and MC5R, besides its agonist activity on the S1PR1 receptor. Indeed, we explored the binding of fingolimod to hMC1R and hMC5R, by means of 20 ns molecular dynamics simulations, and compared it with simulation of hMC1R and hMC5R in complex with selective agonists (hMC1R/BMS-470539, hMC5R/PG901 complexes) and antagonists (hMC1R/Agrp, hMC1R/PG20N). Besides the greater predicted affinity for hMC5R (table 1) compared to hMC1R, during 20 ns simulation, fingolimod in complex with hMC1R receptor showed lower and more stable root mean square deviation (RMSD) plot, compared to fingolimod/hMC5R complex (Figures 6A,B). Additionally, fingolimod had a greater number of ligand-protein interactions, specifically stable H-bonds and a water bridge, with hMC1R receptor (Figure 6C) compared to hMC5R complex (Figure 6D).

**FIGURE 7:**
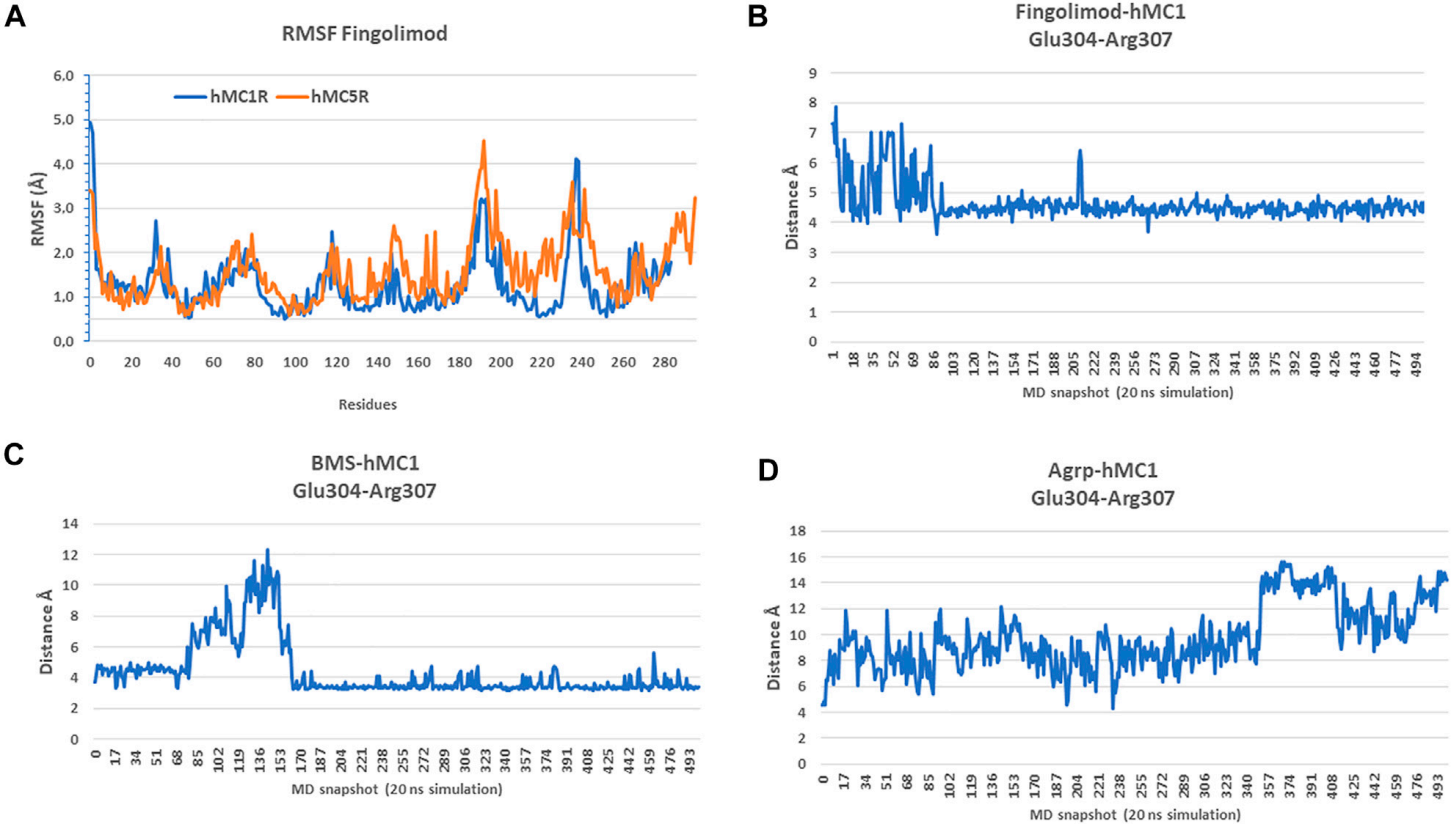
Fingolimod stabilized hMC1R to lower root mean square fluctuations (RMSF), compared to hMC5R. **(A)** RMSF of fingolimod-hMC1R (blue) complex and fingolimod-hMC5R (orange) complex as mean of 20 ns simulations. **(B)** Glu304-Arg307 salt-bridge distance during 20 ns simulation of fingolimod-hMC1R complex. **(C)** Glu304-Arg307 salt bridge distance during 20 ns simulation of BMS-hMC1R complex. **(D)** Glu304-Arg307 salt bridge distance during 20 ns simulation of Agrp-hMC1R complex.

Analysis of root mean square fluctuations (RMSF) showed that fingolimod stabilized hMC1R to lower RMSF values, compared to hMC5R RMSF values (Figure 7A). To confirm that fingolimod works as hMC1R and hMC5R agonist, we tried to shed light on receptor conformational modification induced by fingolimod, comparing salt-bridges of fingolimod/hMC1R and hMC5R complexes with correspondent salt-bridges in validated agonists and antagonists/hMC1R and hMC5R complexes. Data about salt-bridges at VIII of hMC1R, the amphipathic helix of GPCRs parallel to the cytosolic side of lipid membrane, have strengthened the hypothesis and the experimental results of the study hereby presented (Figures 7B–D). In fact, fingolimod bound to hMC1R (Figure 7B), during 20 ns simulation, stabilized to 4Å distance the salt-bridge between Glu304 and Arg307, similarly to BMS/hMC1R complex (Figure 7C). On the contrary, the hMC1R antagonist AGRP destroyed the Glu304-Arg307 salt-bridge (13 ± 0.5 Å, Figure 7D), during the 20 ns simulation. Unfortunately, salt-bridges analysis for hMC5R complexes gave inconclusive results.

**FIGURE 8:**
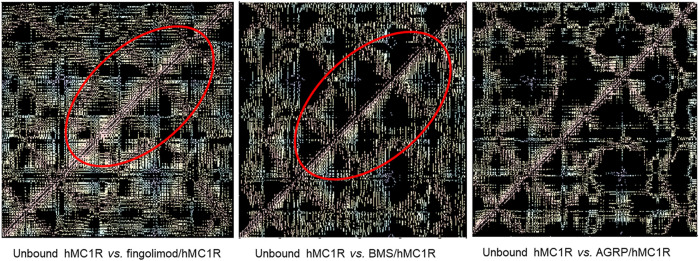
Differences in residue-residues contact maps in hMC1R bound to fingolimod and selective agonist (BMS) and antagonist (AGRP).

**FIGURE 9:**
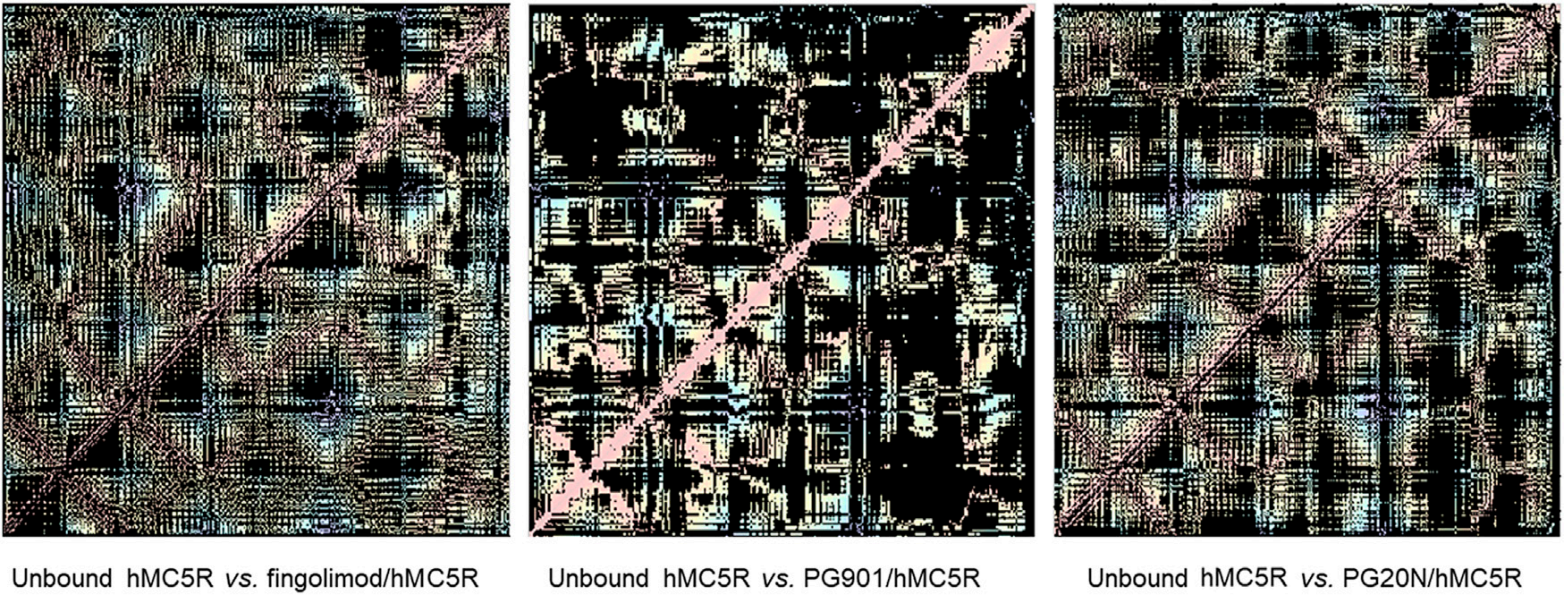
Differences in residue-residues contact maps in hMC5R bound to fingolimod and selective agonist (PG901) and antagonist (PG20N).

As regards as overall conformational changes in hMCxR receptors, upon binding with agonists and antagonists, we built residue-residue contact maps. These contact maps were further analyzed to analyze the receptor conformational changes (i.e., differences between contact maps of unbound receptor compared to ligand-hMCxR complexes), by means of a web application (fuzzy logic algorithm for image differences analyses). We found that fingolimod induced in hMC1R a pattern of residue-residues interactions (i.e., conformational modification), very close to conformational changes induced by the selective hMC1 agonist BMS. The pattern of fingolimod-hMC1R complex was totally different from conformational modifications induced by the antagonist AGRP on hMC1R (Figure 8). These results are in accordance with data on salt-bridges in hMC1R complexes (Figures 7B–D). Contact map modifications (i.e., conformational changes) in hMC5R upon binding with fingolimod, PG901 (agonist) and PG20N (antagonist), gave ambiguous information (Figure 9). Specifically, contact maps on hMC5R upon binding with fingolimod gave a pattern of interactions different from conformational changes induced by PG901 and PG20N. Indeed, we can state that fingolimod would be a hMC5R agonist, only on the basis of *in vivo* pharmacological data.

## Discussion

Diabetic retinopathy, the most common complication of diabetes, is the leading cause of blindness in working-age adults (Cheloni et al., 2019). The role of chronic low-grade retinal inflammation in DR etiopathogenesis has been evidenced by several clinical and preclinical studies (Rübsam et al., 2018; Platania et al., 2019a; Lazzara et al., 2019; Lazzara et al., 2020; Trotta et al., 2019; Trotta et al., 2021; Bonfiglio et al., 2020; Rossi et al., 2021). Indeed, DR patients have shown high serum and ocular levels of pro-inflammatory cytokines and chemokines, such as interleukin 1β (IL-1β), interleukin 6 (IL-6), interleukin 8 (IL-8), Tumor Necrosis Factor α (TNF-α), and Monocyte Chemoattractant Protein-1 (MCP-1), transforming growth factor β (TGFβ) (Boss et al., 2017; Wu et al., 2017; Bonfiglio et al., 2020). The increase of these inflammatory mediators has been proposed to contribute to early neurovascular retinal dysfunction (Vujosevic and Simó, 2017; Rübsam et al., 2018; Fu et al., 2020). Particularly, by acting as a pro-inflammatory mediator, VEGFA plays a critical role in DR pathogenesis, by triggering the process of vascular proliferation (Zhao and Singh, 2018; Aguilar-Cazares et al., 2019). Particularly, VEGFA acts on VEGFR1, which generates vascular sprouting, and on VEGFR2, which mediates vao-permeability by activating endothelial nitric oxide synthase (eNOS) (Stuttfeld and Ballmer-Hofer, 2009; Ruszkowska-Ciastek et al., 2014). To this regard, ocular anti-VEGF therapy is the currently gold standard treatment for DR (Cheung et al., 2014), along with intravitreal steroids (Bucolo et al., 2018). Therefore, the modulation of the chronic inflammation, targeting several pathways (Bucolo et al., 2005; Shafiee et al., 2011), could be very useful in order to avoid the progression to the late state of DR, characterized by neuronal loss, increased vascular permeability with blood retinal barrier break, macular edema and finally retinal ischemia and neovascularization (Duh et al., 2017). To this regard, we have previously reported an emerging role of melanocortin receptors subtypes 1 and 5 (MC1R and MC5R), which when activated are able to counteract the retinal pro-inflammatory *milieu* induced by diabetes. In particular, MC1R and MC5R activation restored the levels of manganese-dependent superoxide dismutase (MnSOD) and glutathione peroxidase (GPx) antioxidant enzyme levels in primary retinal cell cultures exposed to high-glucose concentration, reducing pro-inflammatory cytokines and chemokines and consequently preserving photoreceptor integrity (Maisto et al., 2017). Moreover, MC1R and MC5R agonists in diabetic mice reduced DR damage by increasing retinal occludin levels, leading to polarization of M2 macrophages levels and reducing retinal VEGF content (Rossi et al., 2021). Interestingly, melanocortin system has been recently shown to interact with S1P1Rs expressed by hypothalamic neurons in rodents. Particularly, a strong positive correlation was found among hypothalamic S1PR1 mRNA and MC3R and MC4R receptors (Silva et al., 2014). S1PRs modulate different cell functions such as proliferation, migration, angiogenesis, chemotaxis, and immune cell trafficking (Sharma et al., 2013). Particularly, 5 subtypes of S1PRs (S1P1–5) have been identified in humans. These G-protein coupled receptors are differentially expressed in various tissues and cell types, such as endothelial cells, T cells, B cells, macrophages, astrocytes, and neurons (Mehling et al., 2011; Bikbova et al., 2015). It has been demonstrated that, after oral administration, fingolimod is phosphorylated in the central nervous system and binds to S1P1R, S1P3R, S1P4R, and S1P5R with an affinity comparable to the affinity of sphingosine 1-phosphate (S1P) (Mehling et al., 2011). S1P1R is localized also on retinal neurons (Bikbova et al., 2015) and was found to be involved in cytokine production through signal transducer activator transcription 3 (STAT3), and it is also able to induce NOD-like receptor protein 3 (NLRP3) inflammasome, a multiprotein complex activated during diabetic retinal damage (Trotta et al., 2019; Weigert et al., 2019). After binding of FTY720 to S1P1R on lymphocytes and central nervous system (CNS) cells, S1P1R is internalized and degraded (Gräler and Goetzl, 2004), leading to decrease of S1P1R number on the cell surface and impairment of receptor signaling (Chiba et al., 1999). Particularly, fingolimod inhibited the lymphocytes egress from the lymph nodes; therefore, in this condition lymphocytes do not reach the CNS and cannot damage myelin of the nerve fibers (Chiba et al., 1999). Accordingly, S1P1R activation preserved blood brain barrier integrity and blocked peripheral blood mononuclear cells (PBMCs) transmigration (Spampinato et al., 2015; Yamamoto et al., 2017). Consequently, fingolimod action results in a reduction of inflammatory damage mediated by immune cells. Therefore, besides its approved clinical use in patients affected by relapsing-remitting multiple sclerosis (RR-MS) (Mehling et al., 2011), fingolimod effects have been investigated in other immune-inflammatory disorders (Bing et al., 2009; Yoshida et al., 2013). Fingolimod was able to exert an anti-inflammatory action and to increase blood retinal barrier tight junctions expression in a rodent model of DR, by ultimately reducing vascular permeability (Fan and Yan, 2016). To this regard, it is worthy of note that the examination of fingolimod ocular effects in RR-MS patients showed a preserved macular structure and thickness over the time, together with a complete absence of macular edema, even if it is reported as a fingolimod side effect (Fruschelli et al., 2019; d’Ambrosio et al., 2020; Rossi et al., 2020). In our study, a virtual screening approach evidenced that fingolimod, along with other FDA already approved drugs, can bind with good-predicted affinity to melanocortin receptors MC1R and MC5R. Therefore, we then tested fingolimod in an *in vivo* model of DR. Although our DR animal model shows some limitations in evidencing marked changes of retinal vascularity by FA evaluations, since it reproduces only alterations of DR early stages such as vascular caliber irregularity or microaneurysms, conversely immunohistochemical analysis showed a specific modulation of VEGFR1 and VEGFR2 expression, with consequent alterations in retinal neovascularization process, as evidenced by the increase of retinal VEGFA levels. Overall, diabetic C57BL/6J mice treated with fingolimod exhibited a reduction of retinal angiogenesis. Particularly, FA evaluations did not evidence any retinal vessel size irregularity in diabetic mice treated with fingolimod, which led to a reduced VEGFR1 and VEGFR2 retinal staining, compared to untreated diabetic mice. Also, retinal VEGFA levels were reduced by fingolimod treatment. This protective effect was less evident in mice receiving fingolimod combined with MC1R antagonist, showing an irregular retinal vessel size, which did not significantly change over time. Similarly, the combination of MC5R antagonist caused a slight progressive thinning of the vascular caliber. Furthermore, although VEGFR1 and VEGFR2 along with retinal VEGFA content were reduced in mice treated with fingolimod and MC1R or MC5R antagonists compared to the diabetic group, they were significantly higher when compared to diabetic mice treated with fingolimod alone. These results from our *in vivo* pharmacological study suggest that fingolimod acts as an agonist of MC1R and MC5R, as evidenced by our preliminary in-silico (virtual screening of FDA approved drugs) analysis. Particularly, we found that a similar trend was found in diabetic mice co-treated with fingolimod and a selective S1P1R antagonist. This may indicate that fingolimod influences independently melanocortin and SP1 pathways in the retina. These data were further confirmed through molecular dynamics simulations, showing that fingolimod stably binds to hMC1R and hMC5R. Structural analysis of simulation of hMC1R bound to fingolimod supported that fingolimod works as hMC1R agonist, similarly to the selective hMC1R agonist BMS-470539. Data on hMC5R molecular dynamics simulations are less straightforward compared to simulations on MC1R, and fingolimod effects on hMC5R structure are totally different from either antagonist or agonists effects. The present findings highlight that fingolimod is worthy of further pharmaceutical development such as optimization of drug formulations for ocular drug delivery (Conti et al., 1997; Platania et al., 2019b). In conclusion, despite the limitations of our experimental DR model, our data provided evidence that fingolimod exerted anti-angiogenic activity not only through the S1P1 receptor, but also activating MC1R and MC5R, confirming that these GPCRs are intriguing pharmacological targets to handle DR.

## Data Availability

The original contributions presented in the study are included in the article/Supplementary Material, further inquiries can be directed to the corresponding author.
